# Soluble lytic transglycosylase SLT of *Francisella novicida* is involved in intracellular growth and immune suppression

**DOI:** 10.1371/journal.pone.0226778

**Published:** 2019-12-26

**Authors:** Takemasa Nakamura, Takashi Shimizu, Akihiko Uda, Kenta Watanabe, Masahisa Watarai

**Affiliations:** 1 Joint Faculty of Veterinary Medicine, Laboratory of Veterinary Public Health, Yamaguchi University, Yamaguchi, Japan; 2 Department of Veterinary Science, National Institute of Infectious Diseases, Shinjuku, Tokyo, Japan; New York Medical College, UNITED STATES

## Abstract

*Francisella tularensis*, a category-A bioterrorism agent causes tularemia. *F*. *tularensis* suppresses the immune response of host cells and intracellularly proliferates. However, the detailed mechanisms of immune suppression and intracellular growth are largely unknown. Here we developed a transposon mutant library to identify novel pathogenic factors of *F*. *tularensis*. Among 750 transposon mutants of *F*. *tularensis* subsp. *novicida* (*F*. *novicida*), 11 were isolated as less cytotoxic strains, and the genes responsible for cytotoxicity were identified. Among them, the function of *slt*, which encodes soluble lytic transglycosylase (SLT) was investigated in detail. An *slt* deletion mutant (Δ*slt*) was less toxic to the human monocyte cell line THP-1 vs the wild-type strain. Although the wild-type strain proliferated in THP-1 cells, the number of intracellular Δ*slt* mutant decreased in comparison. The Δ*slt* mutant escaped from phagosomes during the early stages of infection, but the mutant was detected within the autophagosome, followed by degradation in lysosomes. Moreover, the Δ*slt* mutant induced host cells to produce high levels of cytokines such as tumor necrosis factor-α, interleukin (IL)-6, and IL-1β, compared with the wild-type strain. These results suggest that the SLT of *F*. *novicida* is required for immune suppression and escape from autophagy to allow its survival in host cells.

## Introduction

*Francisella tularensis*, a gram-negative, facultative intracellular bacterium causes tularemia in humans and animals [1]. Its reservoirs are rabbits and rodents, and it is transmitted to humans via routes such as arthropod bites and direct contact with infected animals [2]. *F*. *tularensis* is easily aerosolized and causes disease in humans at only 10 colony-forming units (CFUs) [3]. Therefore, *F*. *tularensis* is considered a potential biological weapon and, as such, is considered a category-A bioterrorism agent [4].*F*. *tularensis* comprises the subspecies *tularensis* (also called type A), *holarctica* (type B), *mediasiatica*, and *novicida*. Among them, only *F*. *tularensis* subsp. *tularensis* and *F*. *tularensis* subsp. *holarctica* are highly virulent for humans and cause tularemia [4]. Although *F*. *tularensis* subsp. *novicida* (*F*. *novicida*) exhibits low virulence in humans, it is a facultative intracellular pathogen that replicates within macrophages and is pathogenic for mice. Moreover, *F*. *novicida* shares considerable homology with highly virulent subspecies. *F*. *novicida* is therefore widely used as a surrogate for the study of *Francisella* [5].

*Francisella* species are ingested through the pseudopod loops of macrophages and incorporated into vacuoles possessing endosomal markers [6, 7]. Subsequently, the bacteria escape from phagosomes and replicate in the cytosol [8]. The *Francisella* pathogenicity island (FPI) is a gene cluster of approximately 30 kb encoding 16–19 open reading frames, which are required for the intracellular growth of *Francisella* [9]. In these FPI members, several genes are homologous to the core genes that encode the constituents of the type VI secretion system (T6SS) [10, 11]. Deletion mutants of these genes persist in the host cell’s cytosol. Therefore, a different mechanism related to intracellular survival may operate. *Francisella* is immunosuppressive through inhibition of the induction of inflammatory cytokines or melanization, and as a consequence, escapes the immune system, allowing it to survive in mammalian and arthropod hosts [12, 13]. However, the mechanisms underlying immune suppression are unknown.

Lytic transglycosylases (LTs) degrade peptidoglycans by cleaving the β-1,4 bond between N-acetylglucosamine and N-acetylmuramic acid [14]. LTs, which are present in gram-negative bacteria, contribute to the remodeling of peptidoglycans and cell division. LTs are also required for the assembly of macromolecular complexes such as flagella, pili, and secretion systems larger than the size of peptidoglycan pores [15]. Although LTs are closely associated with the virulence of certain bacteria [16], the function of LTs in *Francisella* is unknown.

Here we constructed a transposon mutant library and determined that soluble lytic transglycosylase (SLT) is a novel pathogenic factor of *F*. *novicida*. We showed here that SLT was associated with intracellular growth and immunosuppressive activity, independent of the type VI secretion system (T6SS).

## Materials and methods

### Bacterial strains and culture conditions

*F*. *novicida* U112 was obtained from the Pathogenic Microorganism Genetic Resource Stock Center (Gifu University). *F*. *novicida* was cultured aerobically at 37 °C in a chemically defined medium (CDM) [17] or in brain heart infusion broth (Becton, Dickinson and Company, Franklin Lakes, NJ) supplemented with cysteine (BHIc) [18] and containing 1.5% agar (Wako Laboratory Chemicals, Osaka, Japan). *Listeria monocytogenes* strain EGD was cultured in BHI broth. Bacterial concentrations were adjusted according to the optical density (OD_595_) of the culture medium.

### Cell culture

THP-1 cells (human monocytic cell line) and J774 cells (murine macrophage-like cell line) were grown in RPMI 1640 medium (Sigma-Aldrich, St. Louis, MO) supplemented with 10% heat-inactivated fetal bovine serum at 37 °C in an atmosphere containing 5% CO_2_.

### Cytotoxicity assay

THP-1 cells (4 × 10^5^ cells/well) were incubated in a 12-well or 48-well tissue culture plate with 200 nM phorbol myristate acetate (PMA) for 48 h. *F*. *novicida* strains were used at multiplicity of infection (MOI) of 0.5 or 0.01. The plates were then centrifuged for 10 min at 300 × *g* and incubated for the indicated time. Subsequently, the cells were washed three times with RPMI1640 medium, and extracellular bacteria were killed via exposure to gentamicin (50 μg/ml) for 1 h. To measure lactate dehydrogenase (LDH), the cells were incubated in fresh medium at 37 °C for the indicated time. The release of LDH into supernatants was measured using an LDH Cytotoxicity Detection Kit (Takara Bio, Shiga, Japan).

### Plasmid construction, transformation, and transfection

S1 Table shows the primer sets and templates used to construct plasmids. PCR was performed using KOD-Plus-Neo (Toyobo, Osaka, Japan), and ligation was performed using Ligation High Ver. 2 (Toyobo) or an In-Fusion HD Cloning Kit (Takara Bio). Plasmids were used to transform *F*. *novicida* via cryo-transformation [19]. Briefly, bacterial cells were suspended in transfer buffer (0.2 M MgSO4, 0.1 M Tris acetate [pH 7.5]) with 1 μg of plasmid DNA. The bacterial cells were frozen in liquid nitrogen, thawed at room temperature, cultured in CDM, collected, and cultured on BHIc plates containing 50 μg/ml kanamycin or 2.5 μg/ml chloramphenicol.

### Construction of a transposon mutant library

The transposon mutant library was constructed using the Ez-Tn5 transposon system (Epicentre, Madison, WI). The MCS of pMOD3 was digested using Hind III and EcoRI, and the kanamycin resistance cassette of pKEK1140 [20] was ligated to these sites to generate pMOD3-FtKm. The transposon moiety of pMOD3-FtKm was amplified using PCR, purified, mixed with transposase according to the instruction manual, and then used to transform *F*. *novicida* via cryo-transformation. Transformed bacteria were cultured on BHIc plates containing 50 μg/ml kanamycin.

### Sequence analysis of transposon mutants

pMOD3 harbors the R6Kγ origin of replication of *Escherichia coli*. The genomes of *F*. *novicida* transposon mutants were purified using a PureLink Genomic DNA Mini Kit (Thermo Fisher, Waltham, MA) and digested with a combination of Xho I, BglII, EcoRI, SalI, NotI, and BamHI. The ends of the digested DNAs were then blunted using a DNA Blunting Kit (Takara Bio) and ligated using Ligation High Ver. 2 (Toyobo). The ligated DNA was used to transform One Shot PIR1 Chemically Competent *E*. *coli* (Thermo Fisher). The transformed *E*. *coli* were selected for kanamycin resistance, and the plasmid DNAs were purified. Sequence analysis was performed using the primer described in the instruction manual for the Ez-Tn5 transposon system.

### Construction of *F*. *novicida* mutants

The *dotU* homolog (FTN_1316) deletion mutants (Δ*dotU*) of *F*. *novicida* were generated through group-II intron insertion using the TargeTron Gene Knockout System (Sigma-Aldrich) modified for *Francisella* species [20], as previously described [21]. The *slt* (FTN_0496) deletion mutant (Δ*slt*) was generated via homologous recombination using the *Francisella* suicide vector pFRSU [21]. The upstream and downstream regions of *slt* (1.5 kbp each) were cloned into the BamHI site of pFRSU to generate pFRSU-slt. pFRSU-slt (1 μg) was used to transform *F*. *novicida*, and the cells were cultured on BHIc plates containing 50 μg/ml kanamycin. Isolated bacteria were cultured in CDM without antibiotics for 6 h and then plated on BHIc plates containing 5% sucrose. Deletion of the *slt* gene was confirmed via PCR.

### GFP-, mCherry-, and SLT-expressing *F*. *novicida* strains

The GFP- and mCherry-expressing plasmids pOM5-GFP and pOM5-mCherry were constructed according to published procedures [21]. The chromosomal *slt* gene with its native promoter region (200 bp upstream) from the *F*. *novicida* was cloned into pOM5 to generate pOM5-SLT. pOM5-GFP, pOM5-mCherry, and pOM5-SLT were used to transform wild-type *F*. *novicida* or the Δ*slt* mutant of *F*. *novicida* via cryo-transformation.

### Intracellular growth assay

THP-1 cells (4 × 10^5^ cells/well) were incubated in a 48-well tissue culture plate with 200 nM PMA for 48 h. *F*. *novicida* strains were added at MOI = 1. Plates were centrifuged for 10 min at 300 × *g* and incubated for 1 h at 37 °C. The cells were washed three times with RPMI1640 medium, and extracellular bacteria were killed using gentamicin (50 μg/ml) treatment for 1 h. The cells were incubated in fresh medium at 37 °C for the indicated time. Cells were incubated in the presence of 5 mM 3-methyladenine (3-MA) (Wako Laboratory Chemicals) for the indicated time. To measure intracellular growth, the cells were washed with phosphate-buffered saline (PBS), and then lysed with 0.1% Triton X-100 in CDM. The number of CFUs on BHIc plates was determined via plating serial dilutions of cultures.

### Fluorescence microscopy

THP-1 cells (4 × 10^5^ cells/well) on 12-mm glass coverslips in 48-well tissue culture plates were incubated with 100 nM PMA for 48 h. THP-1 cells were infected with *F*. *novicida* strains and incubated for the indicated times. To visualize lysosomes, cells were stained with LysoTracker Red (Thermo Fisher) according to the source’s instruction manual. To detect LC3, cells were fixed with 4% paraformaldehyde at room temperature for 30 min and permeabilized with 100 μg/ml digitonin for 5 min. Cells were treated with an anti-LC3 antibody (PM036, 1:100, Medical & Biological Laboratories, Nagoya, Japan) and stained using an Alexa Fluor 555-conjugated anti-rabbit IgG (ab150078, 1:1000, Abcam, Cambridge, UK). To detect lysosomal-associated membrane protein 1 (LAMP-1), cells were fixed using the PLP Solution Set (Wako Laboratory Chemicals) containing 5% sucrose for 1 h at 37 °C and then permeabilized using cold methanol for 10 s. The cells were treated with an anti-LAMP-1 antibody (ab25245, 1:100, Abcam) and stained with FITC-conjugated anti-rat IgG (1:1000, Abcam). A FluoView FV100 confocal laser scanning microscope (Olympus, Tokyo, Japan) was used to acquire images of the cells.

### T6SS secretion assay

The T6SS secretion assay was performed according to a published method [22]. To delete the endogenous β-lactamase gene (*bla*, FTN_1072), upstream and downstream sequences of *bla* (1.5 kbp each) were cloned into the BamHI site of pFRSU to generate pFRSU-bla, which was used to transform wild-type, Δ*slt*, or Δ*dotU* mutants to generate Δ*bla*, Δ*slt*Δ*bla*, and Δ*dotU*Δ*bla* mutants. The *iglC* gene (FTN_1322) of *F*. *novicida* encoding the T6SS effector protein was cloned using pOM5, as described above, and the ampicillin resistance gene (*ampR*) derived from pCMV-HA-N (Takara Bio) was cloned downstream of IglC to generate pOM5-IglC-AmpR. To express the fusion protein of IglC and AmpR (IglC-AmpR), pOM5-IglC-AmpR were used to transform the Δ*bla*, Δ*slt*Δ*bla*, or Δ*dotU*Δ*bla* mutant. THP-1 cells (4 × 10^5^ cells/well) on 12-mm glass coverslip in a 48-well tissue culture plate were incubated with 100 nM PMA for 48 h. *F*. *novicida* strains were infected. After incubating for the indicated times, THP-1 cells were treated with the β-lactamase substrate CCF2 AM (Invitrogen, Waltham, MA). CCF2 AM (green fluorescence) was digested by IglC-AmpR that was secreted into the cytosol of THP-1 cells, and β-lactamase activity was detected as blue fluorescence.

### ELISA

THP-1 cells (4 × 10^5^ cells/well) were incubated in a 48-well tissue culture plate with 100 nM PMA for 48 h and then infected with *F*. *novicida* strains, *L*. *monocytogenes*, or treated with 100 ng/ml LPS derived from *E*. *coli* (O55:B5). After incubation for 6 h, the concentrations of tumor necrosis factor (TNF)-α, interleukin (IL)-6, and IL-1β in the supernatants were measured using an ELISA MAX Standard Kit (Biolegend, San Diego, CA) according to the manufacturer’s instructions.

### Statistical analysis

Multiple comparisons using the Bonferroni/Dunnett method or the Student’s *t* test were used to evaluate the significance of differences between groups. *P* <0.05 indicates a significant difference.

## Results

### Identification of genes required for the cytotoxicity of *F*. *novicida*

**Fig 1:**
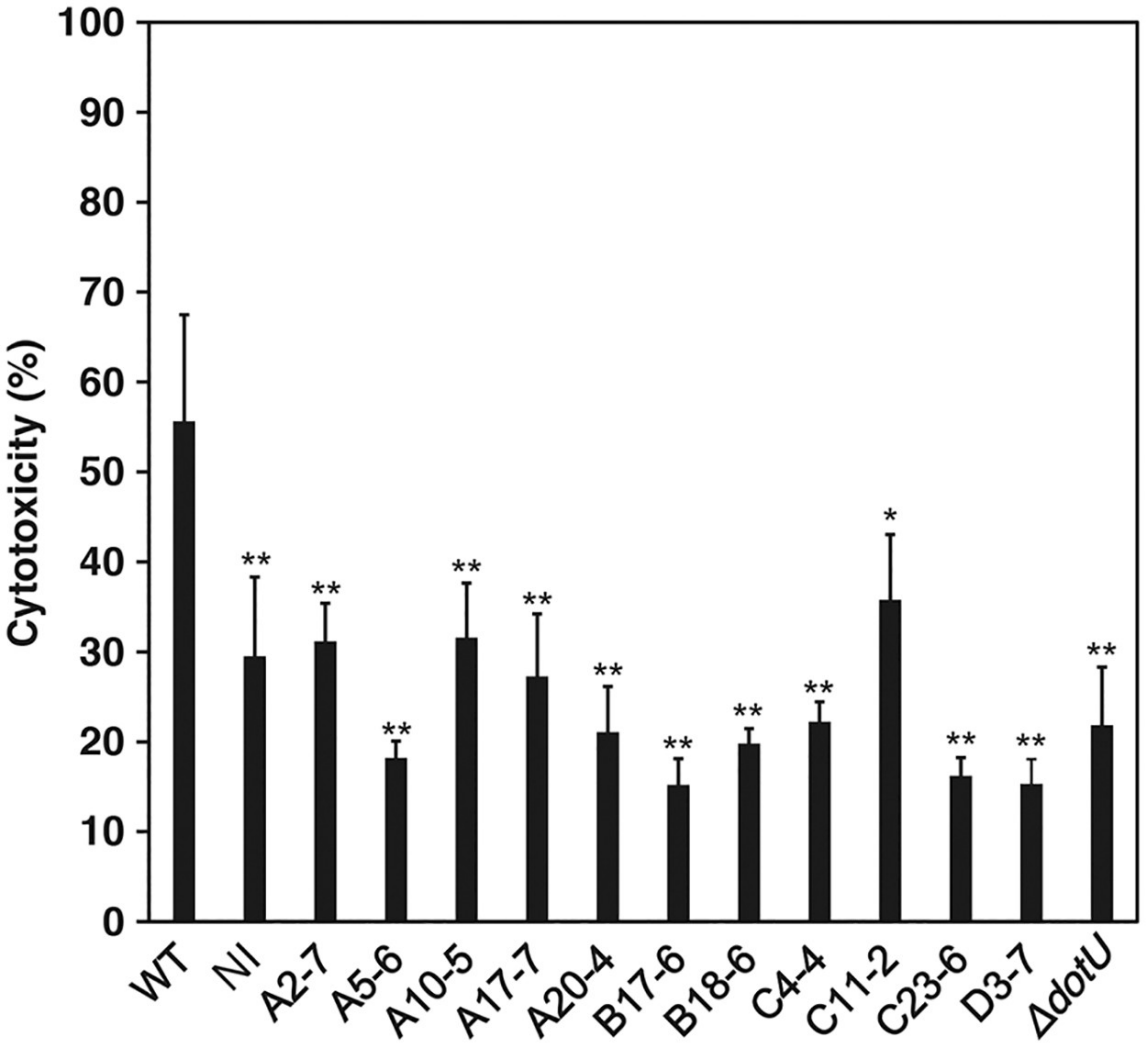
Screening of a transposon mutant library using an LDH cytotoxicity assay. Uninfected THP-1 cells (no infection, NI) or infected with transposon mutants of *F*. *novicida*, MOI = 0.5, were incubated for 24 h, and LDH release was measured. The data represent the averages and standard deviations of three identical experiments. Differences compared with the wild-type strain were analyzed via multiple comparisons and are indicated by asterisks, **P < 0.01, *P < 0.05.

To identify novel virulence factors of *F*. *novicida*, we generated an *F*. *novicida* transposon mutant library. *F*. *novicida* is cytotoxic to the human monocyte cell line THP-1, and cells consequently detach from the culture plate. We therefore performed microscopic observation to screen for mutant strains of *F*. *novicida* lacking cytotoxic activity. Among 750 transposon mutants, 11 mutants were identified as less cytotoxic. To confirm these findings, we performed LDH assays. Compared with the wild-type strain, these mutants caused decreased release of LDH from THP-1 cells (Fig 1). To identify the genes responsible for cytotoxicity, the transposon insertion sites of the mutant strains were sequenced (Table 1). Here we focused on *slt* (FTN_0496) encoding SLT and analyzed its functions.

**Table 1:** Sequence analysis of transposon mutants.

strain	locus_tag	gene name	product
A2-7	FTN_0057	-	major facilitator superfamily (MFS) transport protein
A5-6	FTN_0496	*slt*	soluble lytic murein transglycosylase
A10-5	FTN_1749	-	acyltransferase
A17-7	FTN_0382	-	arsenite-antimonite (ArsB) efflux family
A20-4	FTN_0496	*slt*	soluble lytic murein transglycosylase
B17-6	FTN_1323	*iglB*	intracellular growth locus protein B
B18-6	FTN_0057	-	major facilitator superfamily (MFS) transport protein
C4-4	FTN_0177	*purH*	AICAR transformylase/IMP cyclohydrolase
C11-2	FTN_0569	*recJ*	single-stranded-DNA-specific exonuclease
C23-6	FTN_0141	-	ABC transporter, ATP-binding protein
D3-7	FTN_1159	*ggt*	gamma-glutamyl transpeptidase

### Effect of SLT on intracellular growth and cytotoxicity

**Fig 2:**
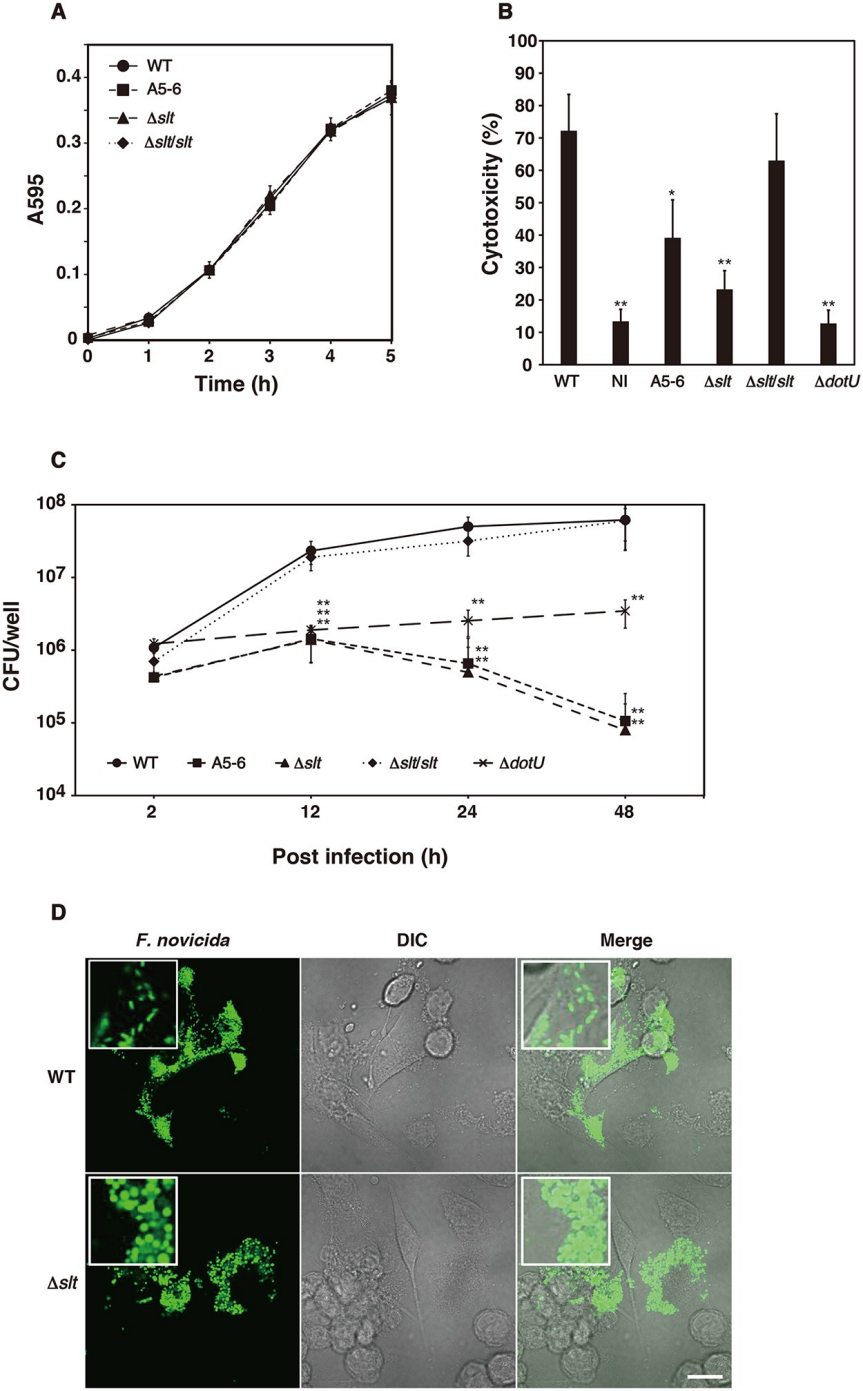
Characteristics of the *slt* deletion mutant. (A) *F*. *novicida* strains were cultured in BHIc medium, and absorbance was measured at A595. (B) Uninfected THP-1 cells (no infection, NI) or THP-1 cells infected with transposons and deletion mutants of *F*. *novicida*, MOI = 0.01, were treated with 50 μg/ml gentamicin for 1 h. Cells were washed and incubated for 48 h, and LDH release was measured. (C) THP-1 cells were infected with *F*. *novicida*, MOI = 1, and were treated with 50 μg/ml gentamicin. Cells were disrupted with 0.1% Triton X-100 and plated on BHIc agar at the indicated times after infection. The data represent the averages and standard deviations of three identical experiments. Differences from the wild-type strain were analyzed via multiple comparison and indicated by asterisks, **P < 0.01, *P < 0.05. (D) THP-1 cells were infected with *F*. *novicida*, MOI = 1, and treated with 50 μg/ml gentamicin for 1 h. The cells were fixed and observed 12 h after infection. Scale bar = 20 μm.

To evaluate the effect of SLT on cytotoxicity, we constructed the *slt* deletion mutant (Δ*slt*) of *F*. *novicida* via homologous recombination. The deletion and transposon mutants of *slt* grew at rates equivalent to those of the wild-type and the complemented (Δ*slt*/*slt*) strains when grown in BHIc medium (Fig 2A). The comma-shape of the Δ*slt* mutant differed from that of the wild-type strain (S1 Fig). The Δ*slt* mutant induced decreased LDH release compared with that of the wild-type strain, whereas the complemented strain released amounts of LDH comparable to that of the wild-type strain (Fig 2B). Deletion of the *dotU* homolog (FTN_1316, Δ*dotU*), a gene encoding a component of the T6SS apparatus, yielded reduced LDH release. To investigate the mechanism of cytotoxicity, we measured the intracellular growth of the Δ*slt* mutant in THP-1 cells. The wild-type strain proliferated intracellularly. In contrast, the number of intracellular Δ*slt* mutant significantly decreased compared with that of the wild-type strain. The number of intracellular Δ*dotU* mutant was maintained (Fig 2C). We next visualized intracellular bacteria using GFP expressing *F*. *novicida* strains. Intracellular wild-type and Δ*slt* mutant were observed 12 h after infection (S2 Fig). However, the larger and spherical shape of the intracellular Δ*slt* mutant differed from that of the wild-type strain (Fig 2D). Because *F*. *novicida* exhibits low virulence in humans and is pathogenic in mice, we investigated the intracellular growth of *F*. *novicida* in the murine macrophage-like cell line J774. Similar to THP-1 cells, the intracellular growth of Δ*slt* mutant was reduced in J774 cells compared with the wild-type strain (S3 Fig). These results suggested that *slt* was required for the cytotoxicity and intracellular growth of *F*. *novicida*.

### Involvement of SLT in escape from phago-lysosomes

To gain further insights into the role of *slt* in the intracellular growth of *Francisella*, we observed the intracellular behavior of the Δ*slt* mutant in THP-1 cells. THP-1 cells were infected with GFP-expressing *F*. *novicida* strains and observed using confocal microscopy. At first, the ability of *F*. *novicida* to escape from phago-lysosome at the early stages of infection (0.5 h, 1 h, and 2 h after infection) was observed using Lysotracker. Colocalization of Lysotracker and the wild-type or Δ*slt* strain was not observed (S4 Fig), suggesting that the wild-type and Δ*slt* strains escaped from the phago-lysosomes.

### Involvement of SLT in recognition by autophagy

**Fig 3:**
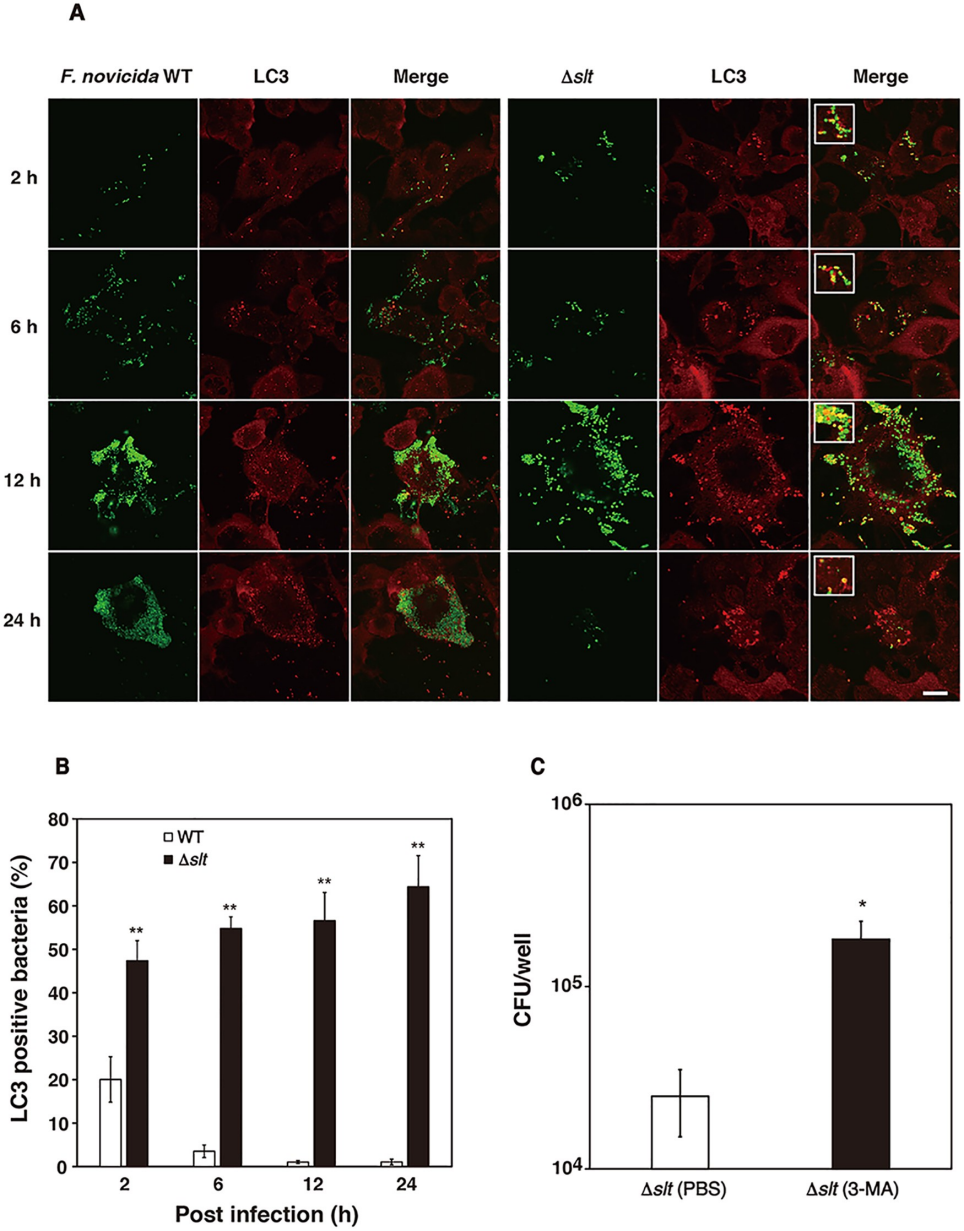
Recognition of *F*. *novicida* strains by autophagosomes. (A) THP-1 cells were infected with *F*. *novicida*, MOI = 1, and treated with 50 μg/ml gentamicin. After infection (2–24 h) cells were treated with an anti-LC3 antibody and stained with Alexa Fluor 555-conjugated anti-rabbit IgG. Scale bar = 20 μm. (B) The ratio of *F*. *novicida* colocalized with LC3 to those that were not was calculated. The data represent the averages and standard deviations of three identical experiments. Differences from the wild-type strain were analyzed via multiple comparison and indicated by asterisks, **P < 0.01. (C) THP-1 cells were infected with *F*. *novicida*, MOI = 1. The cells were treated with 50 μg/ml gentamicin for 1 h, then incubated with 5 mM of 3-MA. They were then disrupted using 0.1% Triton X-100 and plated on BHIc agar 48 h after infection. The data represent the averages and standard deviations of three identical experiments. Differences compared with PBS treatment were analyzed using the Student’s *t* test, *P < 0.05.

We next observed the intracellular behavior of the bacterial strains during the late stages of infection. The wild-type strain proliferated intracellularly from 2 h to 24 h after infection. Consistent with the numbers of intracellular bacteria (Fig 2C), the number of Δ*slt* mutant increased until 12 h after infection, although the number of bacteria decreased 24 h after infection (Fig 3A). The relationship between *F*. *novicida* strains and autophagy was next evaluated. In THP-1 cells infected with the wild-type strain, colocalization of bacteria with the autophagosome marker LC3 was infrequent (Fig 3A). Specifically, 20% of wild-type bacteria colocalized with LC3 after 2 h, and <5% of bacteria were associated with LC3 6–24 h after infection. In contrast, the Δ*slt* mutant colocalized with LC3 throughout infection (Fig 3A) at frequencies ranging from 50%–70% (Fig 3B), indicating that SLT was required for *F*. *novicida* to evade sequestration and destruction via autophagy. To confirm the association between the Δ*slt* mutant and autophagy, THP-1 cells were treated with the autophagy inhibitor 3-MA and infected with *F*. *novicida* strains. In the presence of 3-MA, the intracellular growth of the Δ*slt* mutant significantly increased 48 h after infection (Fig 3C).

### Effect of SLT on the escape of bacterial cells from lysosomes

**Fig 4:**
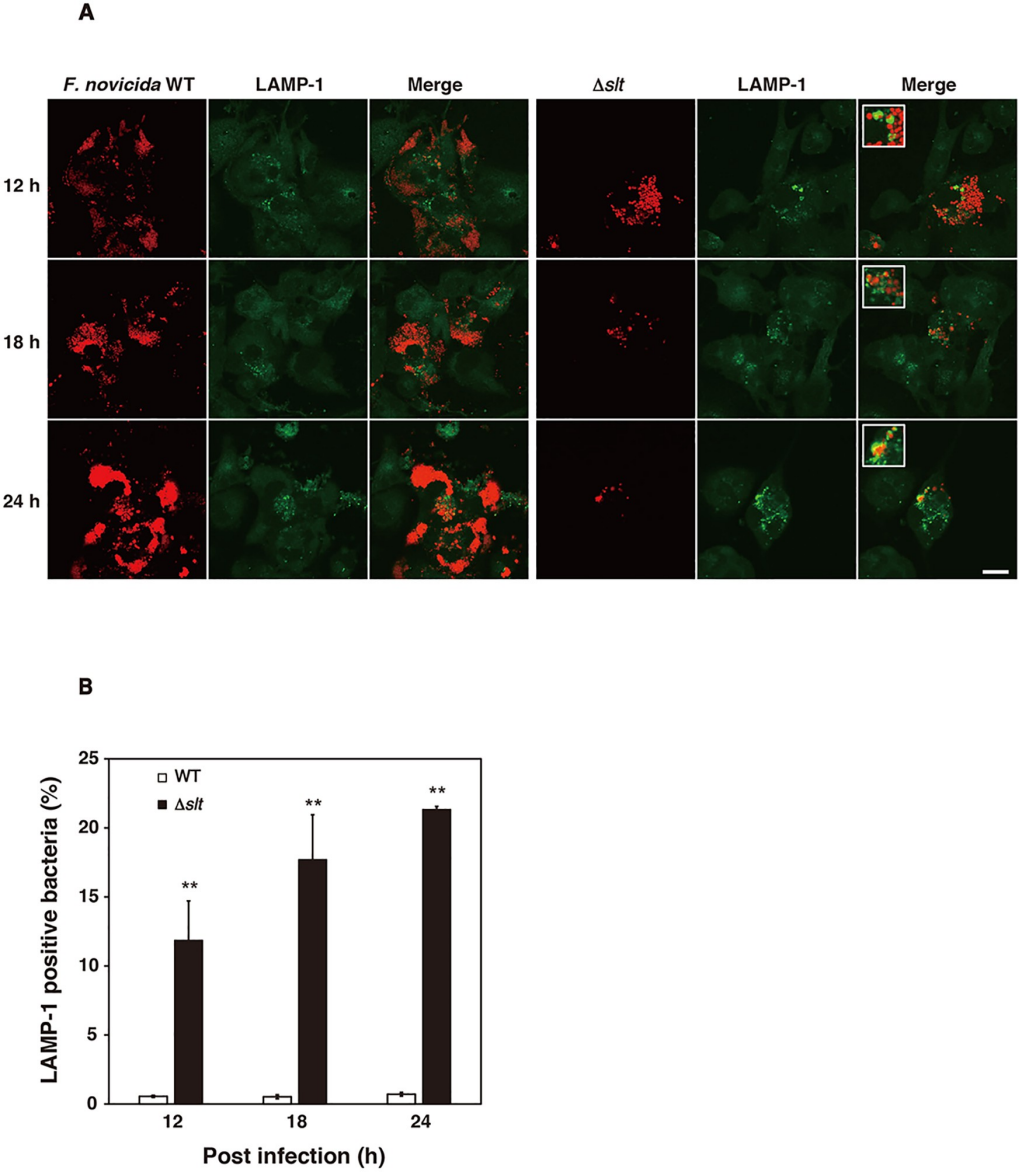
Recognition of *F*. *novicida* strains by lysophagosomes. (A) THP-1 cells were infected with *F*. *novicida*, MOI = 1, and treated with 50 μg/ml gentamicin. Cells were treated with an anti-LAMP-1 antibody and stained with a TRITC-conjugated anti-rat IgG 12 to 24 h after infection. Scale bar = 20 μm. (B) The ratio of *F*. *novicida* colocalized with LAMP-1 was calculated. The data represent the averages and standard deviations of three identical experiments. Differences between the wild-type strain at each time were analyzed using multiple comparisons and are indicated by asterisks, **P < 0.01.

To determine whether the Δ*slt* mutant was digested by lysosomes after capture by autophagosomes, THP-1 cells were infected with the Δ*slt* mutant, and lysosomes were visualized using an antibody against LAMP-1 (Fig 4A). When THP-1 cells were infected with the wild-type strain, intracellular bacterial cells were observed, but few of them colocalized with LAMP-1 (Fig 4A and 4B). In contrast, the Δ*slt* mutant was observed 12 h after infection, although the number decreased 18 h and 24 h after infection (Fig 4A). The ratio of Δ*slt* mutant that colocalized with LAMP-1 increased from 10% to 20% 12–24 h after infection (Fig 4B). These results suggested that *F*. *novicida* escaped degradation by lysosomes in an SLT-dependent manner.

### Relationships between SLT and T6SS

LTs play an important role in forming the T6SS in *E*. *coli* [23]. Therefore, we evaluated the influence of SLT on the activity of the T6SS. To express the T6SS effector protein IglC fused with β-lactamase (IglC-AmpR), *iglC* and *ampR* encoding β-lactamase were cloned into a plasmid (*iglC-ampR*). The β-lactamase activity of the secreted fusion protein was detected using the β-lactamase substrate CCF2 AM (S5 Fig). Wild-type *F*. *novicida* possesses an endogenous β-lactamase gene (*bla*), and consequently, CCF2 AM (green fluorescence) was digested and blue fluorescence was observed. Although β-lactamase activity was not detected when the Δ*bla* mutant was infected, the activity was detected when the Δ*bla* mutant containing *iglC-ampR* (Δ*bla/iglC-ampR*) was infected. The Activity was not detected when the Δ*bla*–Δ*dotU* double-mutant containing *iglC-ampR* (Δ*bla*Δ*dotU*/*iglC-ampR*) was infected. These results suggest that IglC-AmpR was secreted into the cytosol of THP-1 cells through the T6SS. In contrast, the activity was detected when the Δ*bla* and Δ*slt* double-mutant containing *iglC-ampR* (Δ*bla*Δ*slt*/*iglC-ampR*) was infected, indicating that the T6SS was active and effective in the Δ*slt* mutant.

### Relationship between SLT and the immune response

**Fig 5:**
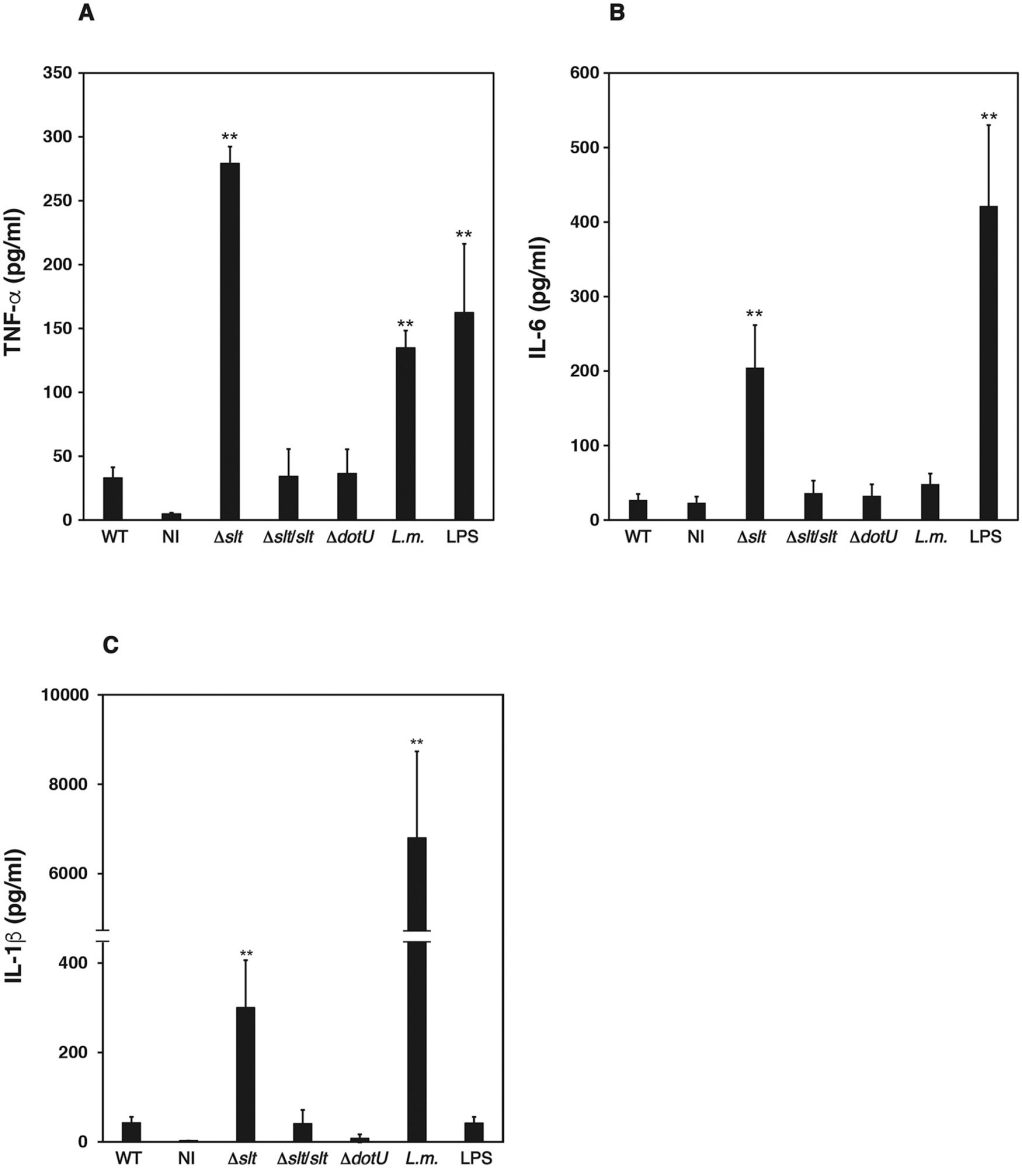
Cytokine induction by *F*. *novicida*. THP-1 cells were treated with 100 ng /ml LPS, and infected (MOI = 1) with *F*. *novicida* or *L*. *monocytogenes*, or incubated with culture medium (no infection, NI). After 6 h of incubation, cell supernatants were collected and the concentrations of TNF-α (A), IL-6 (B), and IL-1β (C) were measured using ELISAs. The data represent the averages and standard deviations of three identical experiments. Differences between the wild-type strain were analyzed using multiple comparisons (A, B) or the Student’s *t* test (C) and are indicated by asterisks,**P < 0.01.

*Francisella* induces immune suppression, and the immune responses to *Francisella* are maintained at relatively low levels compared with those of other bacteria such as *Listeria monocytogenes* [24]. To assess the effect of *slt* on immune responses, we measured the induction of cytokines produced by THP-1 cells. The induction of TNF-α, IL-6, and IL-1β was maintained at relatively low levels compared with infection with *L*. *monocytogenes* or treatment with LPS. In contrast, the Δ*slt* mutant induced high levels of cytokines compared with cells infected with the wild-type and complemented strains (Fig 5). These results indicated that *F*. *novicida* induced immune suppression through an SLT-dependent pathway.

## Discussion

The molecular mechanisms underlying the pathogenicity of *Francisella* species are poorly understood. Here we developed a transposon mutant library of *F*. *novicida* to isolate mutants that were less cytotoxic to THP-1 cells. Among 11 mutants, major facilitator superfamily (MFS) transport protein, intracellular growth locus protein B, and gamma-glutamyl transpeptidase had already been reported as pathogenic factors of *Francisella* [10, 25, 26]. Two strains among the rest of seven had transposon insertions in different positions of the *slt* gene. We reasoned therefore that *slt* was deeply involved in cytotoxicity and focused on the gene encoding SLT. LTs are lytic enzymes of peptidoglycan which create spaces within the peptidoglycan to insert a protein complex such as a secretion system, flagella, or pili into the peptidoglycan or outer bacterial membrane [27–29]. In certain bacterial species, the expression of LTs are upregulated during infection [30]. Moreover, LTs are closely associated with the pathogenicities of bacteria such as *Brucella abortus* or *Helicobacter pylori* [31, 32]. However, the function of the LTs of *Francisella* is not well understood.

*Francisella* are ingested through the pseudopod loops of macrophages and taken up into spacious vacuoles possessing endosomal markers [6, 7]. Subsequently, the bacteria escape from the phagosomes and replicate in the cytosol [8]. During the late stages of infection, the bacteria re-enter the autophagosomes [33, 34]. *F*. *tularensis* subsp. *holarctica* LVS is sequestered by autophagy after escape from phagosomes to the cell cytosol, but the bacteria escape degradation and acquire amino acids from degraded proteins to replicate in LAMP-1 positive autophagosomes, called *Francisella* containing vacuoles [33]. However, infection with highly virulent *F*. *tularensis* subsp. *tularensis* SchuS4 interferes with the autophagic pathway, and only replication-deficient or damaged cytosolic bacteria are captured by autophagosomes and then degraded through the ubiquitin-SQSTM1-LC3 pathway [34]. We showed here, similar to *F*. *tularensis* subsp. *tularensis* SchuS4, that most *F*. *novicida* replicated within the cytosol of THP-1 cells, and only 1%–20% of the bacteria colocalized with the autophagosome marker LC3. The Δ*slt* mutant grew intracellularly until 12 h after infection of THP-1 cells, but the numbers of intracellular bacteria decreased 24 h and 48 h after infection. Although the Δ*slt* mutant did not colocalize with acidic organelles stained with LysoTracker during the early stages of infection (0.5 h–2 h), the mutant colocalized with the autophagosome marker LC3 and the lysosome marker LAMP-1 during the late stages of infection (12 h–48 h after infection). Moreover, the inhibitor of autophagy 3-MA partially restored the intracellular growth of the Δ*slt* mutant. These results might suggest that the Δ*slt* mutant was able to escape from phagosomes, but the bacteria were damaged during their replication in the cell cytosol and were captured by autophagosomes followed by degradation. Meanwhile, the autophagy inhibitor 3-MA failed to completely restore the intracellular growth of Δ*slt* mutant. In addition, only 50% and 20% of the Δ*slt* mutant colocalized with LC3 and LAMP-1, respectively. Therefore, we were unable to exclude the possibility that most of the Δ*slt* mutant failed to escape from their initial phagosomes and were consequently degraded within them. Nevertheless, these findings suggest that SLT is required for the intracellular replication of *F*. *novicida*.

The Δ*slt* mutant exhibited an increased ability to induce the production of inflammatory cytokines such as TNF-α, IL-6, and IL-1β. *Francisella* species suppress inflammatory responses, enabling these bacterial pathogens to survive in the host [12]. Although induction of inflammatory cytokines via *Francisella* infection is suppressed compared with other bacteria such as *L*. *monocytogenes* [24], the detailed mechanisms underlying immune suppression are unknown. Here we showed that the induction of TNF-α and IL-6 production by THP-1 cells infected with wild-type *F*. *novicida* was suppressed compared with that of *L*. *monocytogenes* infection or LPS treatment. However, induction was restored when the cells were infected with the Δ*slt* mutant. The production of inflammatory cytokines such as TNF-α or IL-6 is generally induced by recognition of bacterial components by Toll-like receptors (TLRs) followed by the nuclear translocation of transcription factors such as NF-κB [35]. In host cells infected with *Francisella*, the production of inflammatory cytokines such as TNF-α or IL-6 is induced by recognition of *Francisella* by TLR2, followed by the recognition of *Francisella* DNA by TLR 9 [36]. These results suggest that *F*. *novicida* suppresses TLR2 and TLR9 signaling through SLT-dependent mechanisms.

The induction of IL-1β production was restored in host cells infected with the Δ*slt* mutant. For maturation and release of IL-1β, stimulation of intracellular receptor inflammasomes is required [37]. After expression of the IL-1β precursor, through TLR signaling in host cells infected with *Francisella*, the intracellular recognition of *Francisella* DNA by the AIM2 inflammasome is required for the conversion of the IL-1β precursor to its mature form [38]. However, the activation of inflammasomes by *Francisella* is suppressed compared with that of cells infected with other bacteria [39]. Here we showed that the induction of IL-1β increased compared with cells infected with wild-type bacteria, although the intracellular growth of the Δ*slt* mutant was relatively decreased. These results suggest the possibility that SLT contributes to the suppression of inflammasome signaling.

LTs play an important role in constructing the T6SS in *E*. *coli* [23]. It is therefore possible that intracellular growth or immune suppression by *F*. *novicida* requires the T6SS. However, the T6SS was active in the Δ*slt* mutant. Further, the intracellular behavior of the T6SS apparatus mutant (Δ*dotU*) differed from that of the Δ*slt* mutant, and the number of intracellular bacteria was constant during the late stages of infection (12 h–24 h after infection). These results indicated that the inability of the Δ*slt* mutant to undergo intracellular growth and mediate immune suppression was independent of the T6SS. In bacteria such as *Acinetobacter*, SLT is required to form type-IV pili, which is associated with bacterial pathogenicity [29]. In *F*. *novicida*, proteins associated with pathogenicity such as peptidase or chitin-binding proteins are secreted from the Type-IV pili apparatus [40]. These findings suggest that such secreted proteins may contribute to the intracellular growth or immunosuppressive activity of *F*. *novicida*.

Most recently, it was reported that the SLT of *F*. *novicida* is associated with bacterial pathogenicity for mice [41] through an unknown mechanism. Here we showed that SLT was involved in the intracellular growth of *F*. *novicida* and in its immunosuppressive activity. These findings suggest that in mouse or human macrophage cells, *F*. *novicida* may suppress the induction of immune responses in an SLT-dependent manner, allowing its escape from immune functions such as recognition and degradation via autophagy.

In conclusion, we identified SLT as a new pathogenic factor of *F*. *novicida*. However, the detailed mechanisms of SLT that contribute to intracellular growth and immunosuppressive activity remain to be identified. Highly pathogenic *Francisella* species such as *F*. *tularensis* subsp. *tularensis* harbor *slt*. Therefore, it is critically important to determine the function of SLT, which may provide a basis for understanding the mechanism through which *Francisella* exerts its pathogenicity.

## Supporting information

S1 Table Vectors and primers. (DOCX)

S1 Fig Morphology of *F*. *novicida* Δ*slt*. *F*. *novicida* strains expressing GFP were incubated in BHIc medium containing 5 μg/ml chloramphenicol (OD_595_ = 0.05). Fluorescence and differential interference contrast images of bacteria cells were observed. Scale bar = 10 μm.
(TIF)

S2 Fig Intracellular growth of *F*. *novicida*. THP-1 cells were infected with GFP-expressing *F*. *novicida* strains, MOI = 1, were treated with 50 μg/ml gentamicin for 1 h. Cells were fixed, and actin filaments of infected cells were stained using 100 nM rhodamine phalloidin conjugate 12 h after infection. Serial z-axis images of infected cells were combined into one 3D image and rotated. Scale bar = 20 μm.
(TIF)

S3 Fig Growth of *F*. *novicida* in J774 cells. J774 cells were infected with *F*. *novicida*, MOI = 1, and treated with 50 μg/ml gentamicin for 1 h. The cells were fixed and observed 6–48 h after infection. Scale bar = 20 μm.
(TIF)

S4 Fig Escape of *F*. *novicida* from phagosomes. THP-1 cells were infected with *F*. *novicida*, MOI = 1, and treated with 50 μg/ml gentamicin. Cells were stained with Lysotracker and acidification of phagosomes was visualized 30 min to 2 h after infection. Scale bar = 20 μm.
(TIF)

S5 Fig T6SS secretion assay. Escape of *F*. *novicida* from phagosomes. THP-1 cells were infected with *F*. *novicida* strains expressing an IglC-AmpR fusion protein, MOI = 1, and treated with 50 μg/ml gentamicin. Cells were treated with CCF2 AM 12–24 h after infection. β-lactamase activity was detected as a blue product when CCF2 AM (green) was hydrolyzed. Scale bar: = 200 μm.
(TIF)

## References

[1] Ellis J, Oyston PC, Green M, Titball RW. Tularemia. Clin Microbiol Rev. 2002;15(4):631–46. 10.1128/CMR.15.4.631-646.2002 12364373 PMC126859

[2] Carvalho CL, Lopes de Carvalho I, Ze-Ze L, Nuncio MS, Duarte EL. Tularaemia: a challenging zoonosis. Comp Immunol Microbiol Infect Dis. 2014;37(2):85–96. Epub 2014/02/01. 10.1016/j.cimid.2014.01.002 . 24480622 PMC7124367

[3] McLendon MK, Apicella MA, Allen LA. Francisella tularensis: taxonomy, genetics, and Immunopathogenesis of a potential agent of biowarfare. Annu Rev Microbiol. 2006;60:167–85. Epub 2006/05/18. 10.1146/annurev.micro.60.080805.142126 16704343 PMC1945232

[4] Maurin M. *Francisella tularensis* as a potential agent of bioterrorism? Expert Rev Anti Infect Ther. 2015;13(2):141–4. 10.1586/14787210.2015.986463 . 25413334

[5] Kingry LC, Petersen JM. Comparative review of *Francisella tularensis* and *Francisella novicida*. Front Cell Infect Microbiol. 2014;4:35 10.3389/fcimb.2014.00035 24660164 PMC3952080

[6] Clemens DL, Lee BY, Horwitz MA. Virulent and avirulent strains of *Francisella tularensis* prevent acidification and maturation of their phagosomes and escape into the cytoplasm in human macrophages. Infect Immun. 2004;72(6):3204–17. 10.1128/IAI.72.6.3204-3217.2004 15155622 PMC415696

[7] Clemens DL, Lee BY, Horwitz MA. *Francisella tularensis* enters macrophages via a novel process involving pseudopod loops. Infect Immun. 2005;73(9):5892–902. 10.1128/IAI.73.9.5892-5902.2005 16113308 PMC1231130

[8] Golovliov I, Baranov V, Krocova Z, Kovarova H, Sjöstedt A. An attenuated strain of the facultative intracellular bacterium *Francisella tularensis* can escape the phagosome of monocytic cells. Infect Immun. 2003;71(10):5940–50. 10.1128/IAI.71.10.5940-5950.2003 14500514 PMC201066

[9] Nano FE, Zhang N, Cowley SC, Klose KE, Cheung KK, Roberts MJ, et al A *Francisella tularensis* pathogenicity island required for intramacrophage growth. J Bacteriol. 2004;186(19):6430–6. 10.1128/JB.186.19.6430-6436.2004 15375123 PMC516616

[10] Bröms JE, Sjöstedt A, Lavander M. The role of the *Francisella tularensis* pathogenicity island in type VI secretion, intracellular survival, and modulation of host cell signaling. Front Microbiol. 2010;1:136 10.3389/fmicb.2010.00136 21687753 PMC3109350

[11] Clemens DL, Ge P, Lee BY, Horwitz MA, Zhou ZH. Atomic structure of T6SS reveals interlaced array essential to function. Cell. 2015;160(5):940–51. 10.1016/j.cell.2015.02.005 25723168 PMC4351867

[12] Gillette DD, Curry HM, Cremer T, Ravneberg D, Fatehchand K, Shah PA, et al Virulent Type A Francisella tularensis actively suppresses cytokine responses in human monocytes. Front Cell Infect Microbiol. 2014;4:45 Epub 2014/05/02. 10.3389/fcimb.2014.00045 24783062 PMC3988375

[13] Suzuki J, Uda A, Watanabe K, Shimizu T, Watarai M. Symbiosis with *Francisella tularensis* provides resistance to pathogens in the silkworm. Sci Rep. 2016;6:31476 10.1038/srep31476 27507264 PMC4979039

[14] Dik DA, Marous DR, Fisher JF, Mobashery S. Lytic transglycosylases: concinnity in concision of the bacterial cell wall. Crit Rev Biochem Mol Biol. 2017;52(5):503–42. Epub 2017/06/24. 10.1080/10409238.2017.1337705 28644060 PMC6102726

[15] Scheurwater E, Reid CW, Clarke AJ. Lytic transglycosylases: bacterial space-making autolysins. Int J Biochem Cell Biol. 2008;40(4):586–91. Epub 2007/05/01. 10.1016/j.biocel.2007.03.018 . 17468031

[16] Koraimann G. Lytic transglycosylases in macromolecular transport systems of Gram-negative bacteria. Cell Mol Life Sci. 2003;60(11):2371–88. Epub 2003/11/20. 10.1007/s00018-003-3056-1 . 14625683 PMC11138577

[17] Nagle SC Jr., Anderson RE, Gary ND. Chemically defined medium for the growth of *Pasteurella tularensis*. J Bacteriol. 1960;79:566–71. 14425793 10.1128/jb.79.4.566-571.1960 PMC278733

[18] Mc Gann P, Rozak DA, Nikolich MP, Bowden RA, Lindler LE, Wolcott MJ, et al A novel brain heart infusion broth supports the study of common *Francisella tularensis* serotypes. J Microbiol Methods. 2010;80(2):164–71. 10.1016/j.mimet.2009.12.005 . 20005265

[19] Pavlov VM, Mokrievich AN, Volkovoy K. Cryptic plasmid pFNL10 from *Francisella novicida*-like F6168: the base of plasmid vectors for *Francisella tularensis*. FEMS Immunol Med Microbiol. 1996;13(3):253–56. 10.1111/j.1574-695X.1996.tb00247.x . 8861039

[20] Rodriguez SA, Yu JJ, Davis G, Arulanandam BP, Klose KE. Targeted inactivation of *Francisella tularensis* genes by group II introns. Appl Environ Microbiol. 2008;74(9):2619–26. 10.1128/AEM.02905-07 18310413 PMC2394887

[21] Shimizu T, Otonari S, Suzuki J, Uda A, Watanabe K, Watarai M. Expression of Francisella pathogenicity island protein intracellular growth locus E (IglE) in mammalian cells is involved in intracellular trafficking, possibly through microtubule organizing center. Microbiologyopen. 2019;8(4):e00684 Epub 2018/07/07. 10.1002/mbo3.684 29978561 PMC6460260

[22] Bröms JE, Meyer L, Sun K, Lavander M, Sjöstedt A. Unique substrates secreted by the type VI secretion system of *Francisella tularensis* during intramacrophage infection. PLoS One. 2012;7(11):e50473 10.1371/journal.pone.0050473 23185631 PMC3502320

[23] Santin YG, Cascales E. Domestication of a housekeeping transglycosylase for assembly of a Type VI secretion system. EMBO Rep. 2017;18(1):138–49. Epub 2016/12/07. 10.15252/embr.201643206 27920034 PMC5210162

[24] Putzova D, Panda S, Hartlova A, Stulik J, Gekara NO. Subversion of innate immune responses by Francisella involves the disruption of TRAF3 and TRAF6 signalling complexes. Cell Microbiol. 2017;19(11). Epub 2017/07/27. 10.1111/cmi.12769 . 28745813

[25] Ireland PM, LeButt H, Thomas RM, Oyston PC. A Francisella tularensis SCHU S4 mutant deficient in gamma-glutamyltransferase activity induces protective immunity: characterization of an attenuated vaccine candidate. Microbiology. 2011;157(Pt 11):3172–9. Epub 2011/08/20. 10.1099/mic.0.052902-0 . 21852349

[26] Balzano PM, Cunningham AL, Grassel C, Barry EM. Deletion of the Major Facilitator Superfamily Transporter fptB Alters Host Cell Interactions and Attenuates Virulence of Type A Francisella tularensis. Infect Immun. 2018;86(3). Epub 2018/01/10. 10.1128/IAI.00832-17 29311235 PMC5820938

[27] Zahrl D, Wagner M, Bischof K, Bayer M, Zavecz B, Beranek A, et al Peptidoglycan degradation by specialized lytic transglycosylases associated with type III and type IV secretion systems. Microbiology. 2005;151(Pt 11):3455–67. Epub 2005/11/08. 10.1099/mic.0.28141-0 . 16272370

[28] Hoppner C, Carle A, Sivanesan D, Hoeppner S, Baron C. The putative lytic transglycosylase VirB1 from Brucella suis interacts with the type IV secretion system core components VirB8, VirB9 and VirB11. Microbiology. 2005;151(Pt 11):3469–82. Epub 2005/11/08. 10.1099/mic.0.28326-0 . 16272371

[29] Crepin S, Ottosen EN, Peters K, Smith SN, Himpsl SD, Vollmer W, et al The lytic transglycosylase MltB connects membrane homeostasis and in vivo fitness of Acinetobacter baumannii. Mol Microbiol. 2018;109(6):745–62. Epub 2018/06/10. 10.1111/mmi.14000 29884996 PMC6185781

[30] Cloud-Hansen KA, Peterson SB, Stabb EV, Goldman WE, McFall-Ngai MJ, Handelsman J. Breaching the great wall: peptidoglycan and microbial interactions. Nat Rev Microbiol. 2006;4(9):710–6. Epub 2006/08/09. 10.1038/nrmicro1486 . 16894338

[31] Bao Y, Tian M, Li P, Liu J, Ding C, Yu S. Characterization of Brucella abortus mutant strain Delta22915, a potential vaccine candidate. Vet Res. 2017;48(1):17 Epub 2017/04/06. 10.1186/s13567-017-0422-9 28376905 PMC5381064

[32] Rohde M, Puls J, Buhrdorf R, Fischer W, Haas R. A novel sheathed surface organelle of the Helicobacter pylori cag type IV secretion system. Mol Microbiol. 2003;49(1):219–34. Epub 2003/06/26. 10.1046/j.1365-2958.2003.03549.x . 12823823

[33] Checroun C, Wehrly TD, Fischer ER, Hayes SF, Celli J. Autophagy-mediated reentry of *Francisella tularensis* into the endocytic compartment after cytoplasmic replication. Proc Natl Acad Sci U S A. 2006;103(39):14578–83. 10.1073/pnas.0601838103 16983090 PMC1600002

[34] Chong A, Wehrly TD, Child R, Hansen B, Hwang S, Virgin HW, et al Cytosolic clearance of replication-deficient mutants reveals *Francisella tularensis* interactions with the autophagic pathway. Autophagy. 2012;8(9):1342–56. 10.4161/auto.20808 22863802 PMC3442881

[35] Yamamoto M, Takeda K. Current views of toll-like receptor signaling pathways. Gastroenterol Res Pract. 2010;2010:240365 Epub 2011/01/05. 10.1155/2010/240365 21197425 PMC3010626

[36] Jones CL, Napier BA, Sampson TR, Llewellyn AC, Schroeder MR, Weiss DS. Subversion of host recognition and defense systems by Francisella spp. Microbiol Mol Biol Rev. 2012;76(2):383–404. Epub 2012/06/13. 10.1128/MMBR.05027-11 22688817 PMC3372254

[37] Brewer SM, Brubaker SW, Monack DM. Host inflammasome defense mechanisms and bacterial pathogen evasion strategies. Curr Opin Immunol. 2019;60:63–70. Epub 2019/06/08. 10.1016/j.coi.2019.05.001 . 31174046

[38] Fernandes-Alnemri T, Yu JW, Juliana C, Solorzano L, Kang S, Wu J, et al The AIM2 inflammasome is critical for innate immunity to Francisella tularensis. Nat Immunol. 2010;11(5):385–93. Epub 2010/03/31. 10.1038/ni.1859 20351693 PMC3111085

[39] Dotson RJ, Rabadi SM, Westcott EL, Bradley S, Catlett SV, Banik S, et al Repression of inflammasome by Francisella tularensis during early stages of infection. J Biol Chem. 2013;288(33):23844–57. Epub 2013/07/04. 10.1074/jbc.M113.490086 23821549 PMC3745331

[40] Hager AJ, Bolton DL, Pelletier MR, Brittnacher MJ, Gallagher LA, Kaul R, et al Type IV pili-mediated secretion modulates Francisella virulence. Mol Microbiol. 2006;62(1):227–37. Epub 2006/09/22. 10.1111/j.1365-2958.2006.05365.x . 16987180

[41] Bachert BA, Biryukov SS, Chua J, Rodriguez SA, Toothman RG Jr., Cote CK, et al A Francisella novicida Mutant, Lacking the Soluble Lytic Transglycosylase Slt, Exhibits Defects in Both Growth and Virulence. Front Microbiol. 2019;10:1343 Epub 2019/07/02. 10.3389/fmicb.2019.01343 31258523 PMC6587636

