# Supplementary material for: Soluble lytic transglycosylase SLT of Francisella novicida is involved in intracellular growth and immune suppression
Source: PLoS One. 2019 Dec 26;14(12):e0226778. doi: 10.1371/journal.pone.0226778 (PMC6932806; doi:10.1371/journal.pone.0226778)
Supplement: S1 Table — (DOCX) [file pone.0226778.s001.docx]

| Vector | Primer | Sequence | Template |
| --- | --- | --- | --- |
| pMOD3-FtKm | pMOD3-kanR.FOR | CATCGTGGCCGGATCGATCTTTTGGGTTGTCACTCATCGTATT | pKEK1440 |
|  | pMOD3-kanR.REV | ATTAACCAATTCTGATTAGAAAAACTCATCGAGCATCAAATGAAACT |  |
|  | pMOD3-kanR-vector.FOR | TCAGAATTGGTTAATTGGTTGTAACACTGG | pMOD3 |
|  | pMOD3-kanR-vector.REV | GATCCGGCCACGATGCG |  |
|  |  |  |  |
| pFRSU-slt | slt-Up1.5.FOR | TAGAACTAGTGGATCCTAAAATAGTTTATAAATCCAGTAACAAAATAAAATATAGTCTGAT | *F.novicida* genome |
|  | slt-up1.5.REV | TTTGAATAAATGATTTCAGAATTAGAAAATTATTTTACTAAAAATACTAACAT |  |
|  | slt-down1.5.FOR | AATCATTTATTCAAATTCTTTTAAAAAGAATATTGTAAACCTGATGTGACAAA | *F.novicida* genome |
|  | slt-down1.5.REV | GCAGCCCGGGGGATCCATACTTTTTAATATTCTCATAAGCATATTTTTCAGACCTTGC |  |
|  |  |  |  |
| pFRSU-bla | bla-Up1.5.FOR | TAGAACTAGTGGATCCTCTATATAAACTTTGATAGCTTCTGCGTGGCCT | *F.novicida* genome |
|  | bls-up1.5.REV | CGTACCGCTTTTTAGCTTTTGTATCTAAATAAATAGTAGCTAGAGTTTATATAAGA |  |
|  | bla-down1.5.FOR | CTAAAAAGCGGTACGCCACATTG | *F.novicida* genome |
|  | bla-down1.5.REV | GCAGCCCGGGGGATCCCACAACGTATTAATTCAGTTTCAAAAGTTGAG |  |
|  |  |  |  |
| pOM5-SLT | pOM5-slt.FOR | AGTCTCTTCTCTAGAACAGTTTTTAAAATTAAGTAAGTATATTGTGCTATTGTAATTTAAT | *F.novicida* genome |
|  | pOM5-slt.REV | TGATCTTTTCTACGGTTATTTTCTAAAACTCTGCTTGTCAGATATCTTATAATTCAAGAAAT |  |
|  | pOM5-vector-liner.FOR | CCGTAGAAAAGATCAAAGGATCTTCTTGAGATC | pOM5-IglE |
|  | pOM5-slt-vector.REV | TCTAGAGAAGAGACTGACAAGCTTTTAAAAGACT |  |
|  |  |  |  |
| pOM5-IglC | pOM5-iglC.FOR | AGCAAGGAGAAGTCAATGAGTGAGATGATAACAAGACAACAGGTAACAAGTGG | *F.novicida* genome |
|  | pOM5-iglC.REV | TGATCTTTTCTACGGCTATGCAGCTGCAATATATCCTATTTTAGCAAC |  |
|  | pOM5-vector-liner.FOR | CCGTAGAAAAGATCAAAGGATCTTCTTGAGATC | pOM5-IglE |
|  | pOM5-iglC-vector.REV | TGACTTCTCCTTGCTAGGCCC |  |
|  |  |  |  |
| pOM5-IglC-AmpR | pOM5-iglC-ampR.FOR | TATATTGCAGCTGCAAGTATTCAACATTTCCGTGTCGC | pCMV-HA-N |
|  | pOM5-iglC-ampR.REV | TCTTTTCTACGGCTACCAATGCTTAATCAGTGAGGCACC |  |
|  | pOM5-vector-liner.FOR | CCGTAGAAAAGATCAAAGGATCTTCTTGAGATC | pOM5-IglE |
|  | pOM5-iglC-ampR-vector.REV | TGCAGCTGCAATATATCCTATTTTAGCAACT |  |
