# Supplementary figures and images for: Soluble lytic transglycosylase SLT of Francisella novicida is involved in intracellular growth and immune suppression
Source: PLoS One. 2019 Dec 26;14(12):e0226778. doi: 10.1371/journal.pone.0226778 (PMC6932806; doi:10.1371/journal.pone.0226778)

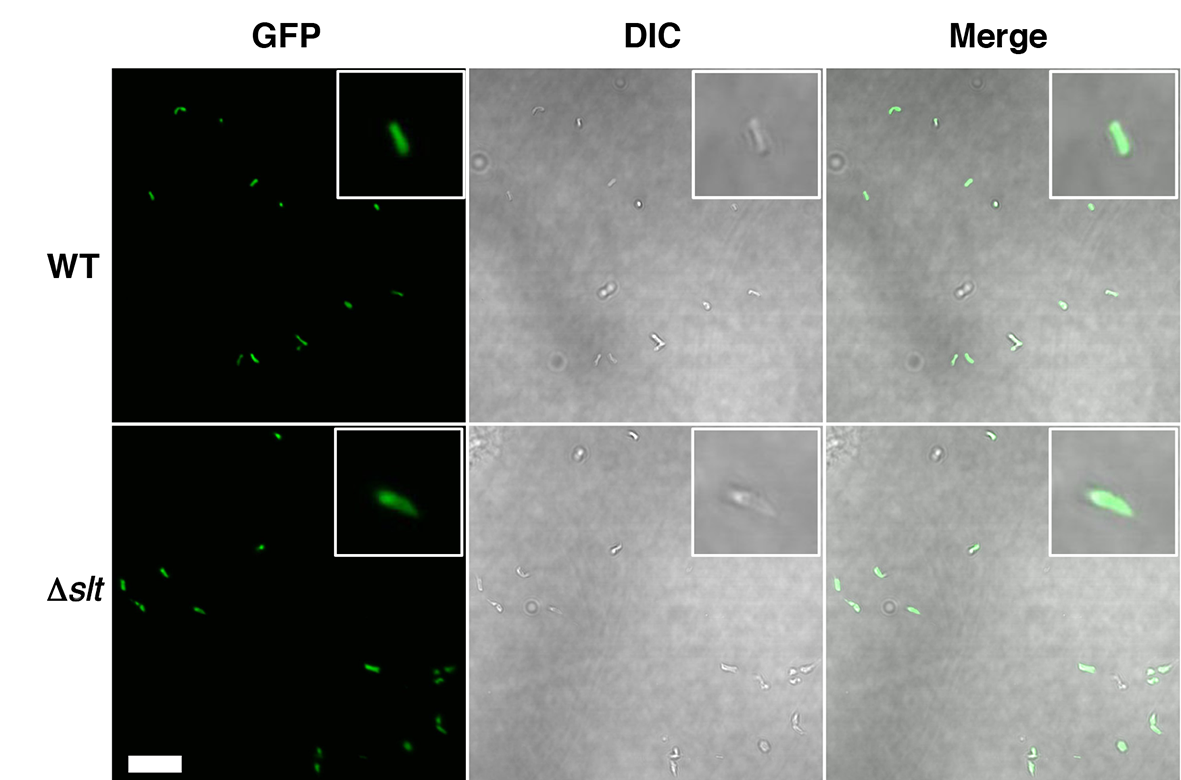

Supplement: S1 Fig — F. novicida strains expressing GFP were incubated in BHIc medium containing 5 μg/ml chloramphenicol (OD595 = 0.05). Fluorescence and differential interference contrast images of bacteria cells were observed. Scale bar = 10 μm. (TIF) [file pone.0226778.s002.tif]

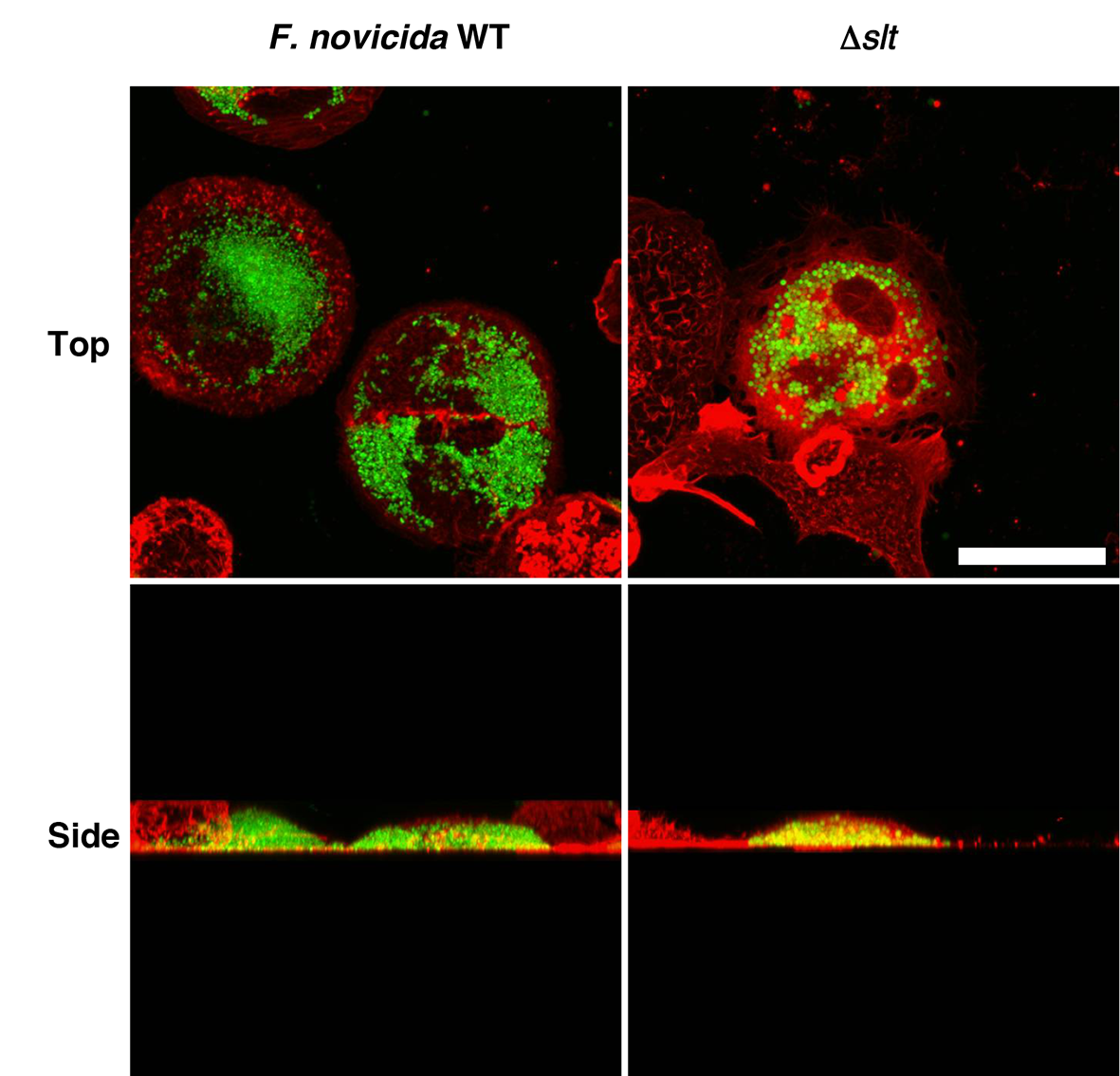

Supplement: S2 Fig — THP-1 cells were infected with GFP-expressing F. novicida strains, MOI = 1, were treated with 50 μg/ml gentamicin for 1 h. Cells were fixed, and actin filaments of infected cells were stained using 100 nM rhodamine phalloidin conjugate 12 h after infection. Serial z-axis images of infected cells were combined into one 3D image and rotated. Scale bar = 20 μm. (TIF) [file pone.0226778.s003.tif]

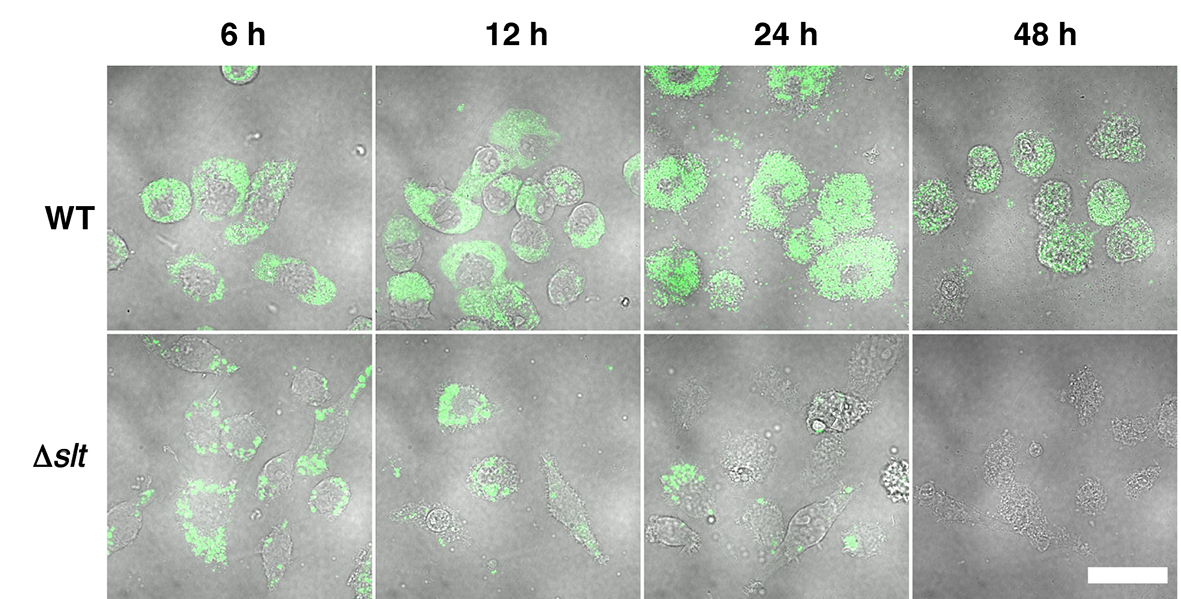

Supplement: S3 Fig — J774 cells were infected with F. novicida, MOI = 1, and treated with 50 μg/ml gentamicin for 1 h. The cells were fixed and observed 6–48 h after infection. Scale bar = 20 μm. (TIF) [file pone.0226778.s004.tif]

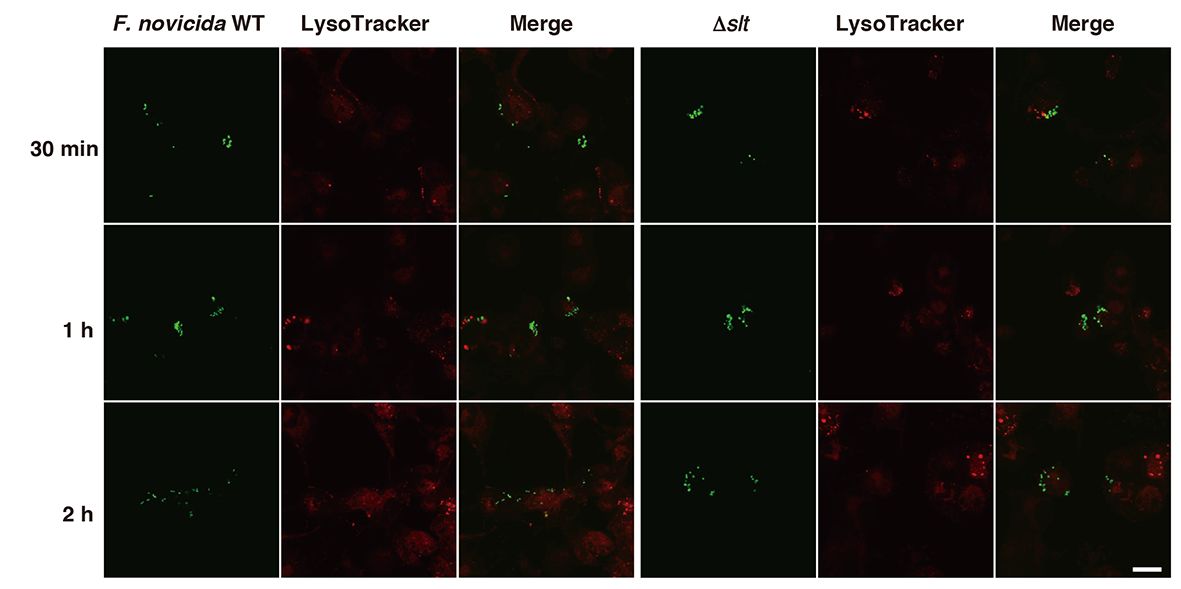

Supplement: S4 Fig — THP-1 cells were infected with F. novicida, MOI = 1, and treated with 50 μg/ml gentamicin. Cells were stained with Lysotracker and acidification of phagosomes was visualized 30 min to 2 h after infection. Scale bar = 20 μm. (TIF) [file pone.0226778.s005.tif]

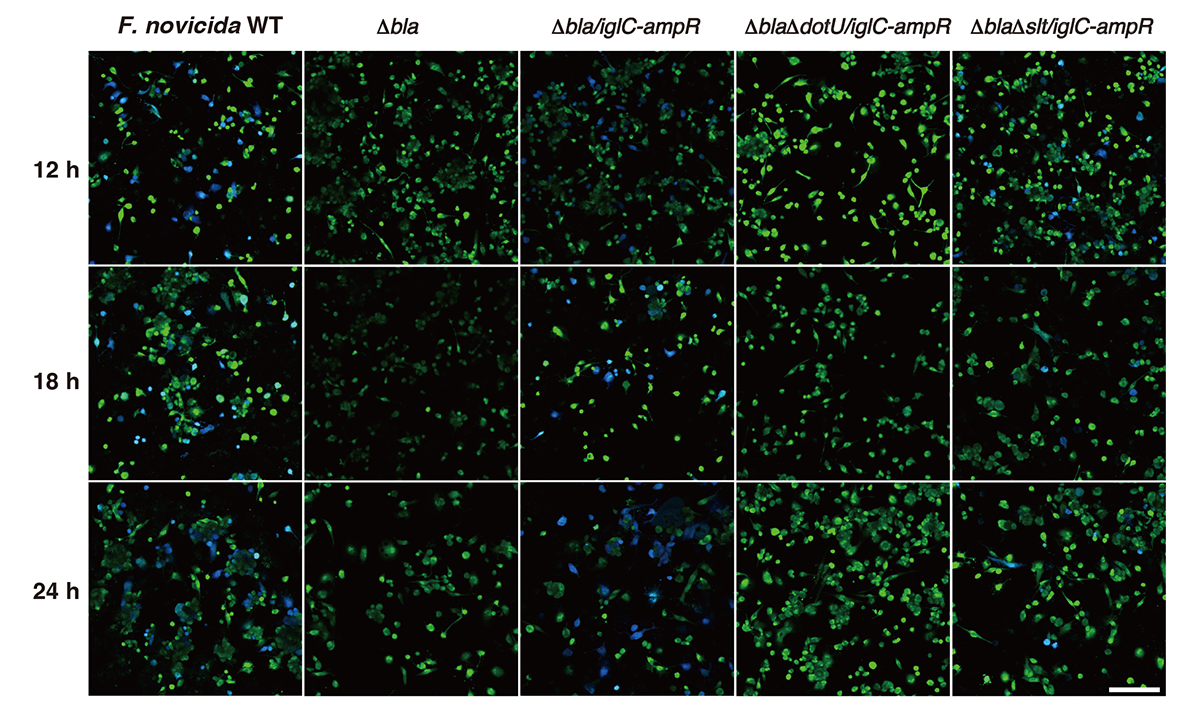

Supplement: S5 Fig — THP-1 cells were infected with F. novicida strains expressing an IglC-AmpR fusion protein, MOI = 1, and treated with 50 μg/ml gentamicin. Cells were treated with CCF2 AM 12–24 h after infection. β-lactamase activity was detected as a blue product when CCF2 AM (green) was hydrolyzed. Scale bar: = 200 μm. (TIF) [file pone.0226778.s006.tif]
